# Serotonin neuromicrobiology: Psychobiotic modulation of the gut–brain axis for mental health

**DOI:** 10.1002/ibra.70028

**Published:** 2026-08-31

**Authors:** Nidhi Mandloi, Aarzoo, Chakkaravarthi Saravanan, Heera Ram, Bhim Pratap Singh

**Affiliations:** ^1^ Department of Postharvest Technology Bioprocessing and Environmental Sciences National Institute of Food Technology Entrepreneurship and Management, Kundli Sonipat Haryana India; ^2^ Department of Interdisciplinary Sciences National Institute of Food Technology Entrepreneurship and Management, Kundli Sonipat Haryana India; ^3^ Department of Zoology Jai Narain Vyas University Jodhpur Rajasthan India

**Keywords:** 5‐hydroxytryptamine, gut–brain axis, psychobiotics, serotonin

## Abstract

The rise in mental health disorders has increased interest in novel natural therapeutic approaches beyond pharmacological interventions. Psychobiotics are live microorganisms that confer mental health benefits and have emerged as a promising intervention targeting the gut–brain axis. Evidence from preclinical studies demonstrated that psychobiotics can restore neurotransmitter balance by modulating pro‐inflammatory cytokines and enhancing the expression of brain‐derived neurotrophic factor. However, significant challenges remain, including the limited availability of clinical studies, determining optimal dosages, and understanding the long‐term benefits of specific psychobiotic strains and their application in food matrices. This review highlights the findings from recent studies to identify psychobiotics' capability on tryptophan metabolism and their role in facilitating the synthesis of serotonin to propose a mechanistic framework for microbe‐driven serotonin. While the eukaryotic pathways of serotonin biosynthesis are well defined, the specific mechanism by which bacteria contribute to this synthesis remains less understood; elucidating this metabolic link is essential. Beyond mechanism, this review evaluates the synergy between psychobiotics and food matrices to showcase the therapeutic approach to address the limitations of current interventions.

## Graphical abstract

An overview of Psychobiotics and Tryptophan metabolism: A serotonin‐kynurenine crosstalk shaping the Gut‐Brain axis. IDO1, Indoleamine 2,3‐dioxygenase 1; 5‐HT3, 5‐hydroxytryptamine type 3; IFN‐γ, Interferon‐gamma; TNF‐α, Tumor necrosis factor‐alpha; IL‐1β, Interleukin‐1 beta; IL‐6, Interleukin 6; Trp, Tryptophan; 5‐HT, 5‐hydroxy‐tryptamine; 5‐HTP, 5‐hydroxy‐tryptophan; SCFAs, Short‐chain fatty acids; TPH1, Tryptophan hydroxylase 1; AADC, Aromatic L‐amino acid decarboxylase; EC, Enterochromaffin cell; AhR, Aryl hydrocarbon receptor; TGR5, Takeda G protein‐coupled receptor 5.

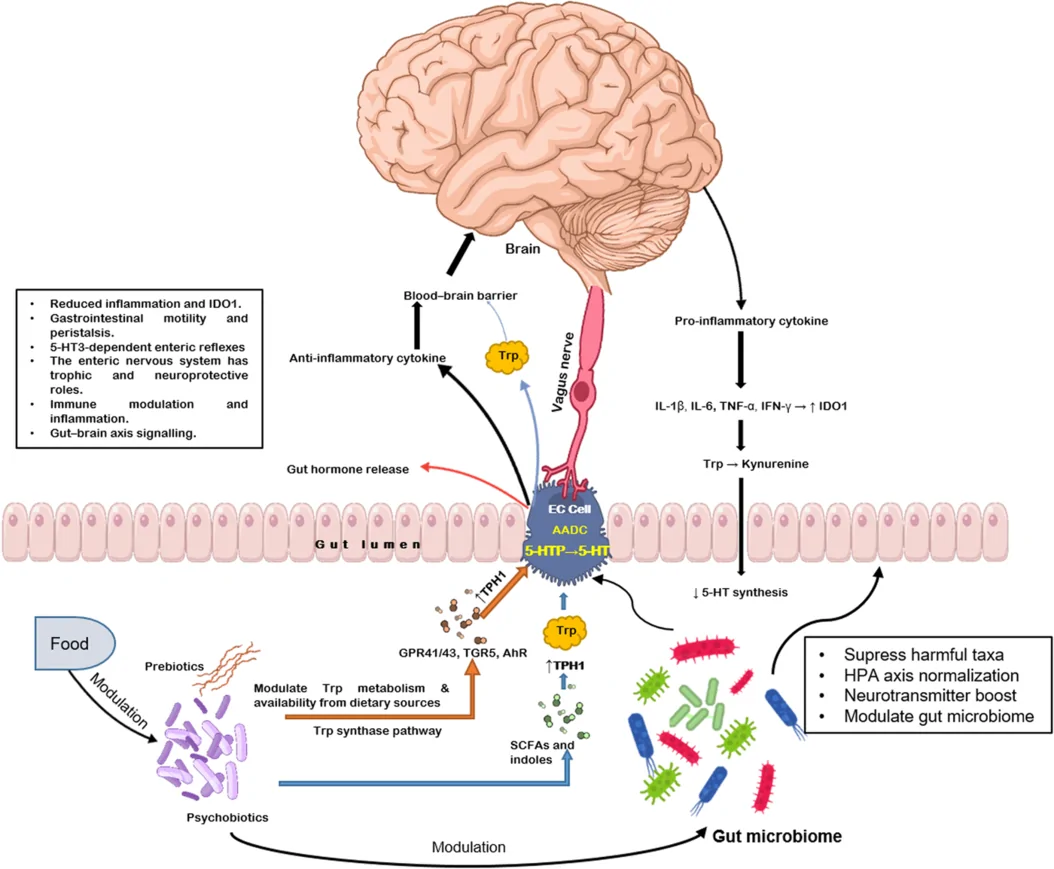

## 1 INTRODUCTION

Over a billion individuals worldwide suffer from mental and addictive disorders, which account for 7% of the global disease burden, and this burden rises as people age.^1^ According to the World Mental Health Report, around 1.095 billion people worldwide, approximately one in seven, are afflicted with mental illnesses, indicating a startling burden of mental health issues. Among these issues, depression and anxiety are the most prevalent disorders, impacting 332 and 359 million people, respectively.^2^ Mental disorders now account for 15.6% of global years lived with disability, making them the leading cause of disability worldwide. Cognitive and developmental disorders also present a growing challenge, particularly in younger populations, with conditions such as autism and attention deficit hyperactivity disorder contributing to early‐onset mental health issues.^3^ Older adults face cognitive decline and depression, while suicide remains a leading cause of death globally.^4^ Despite novel pharmaceutical therapies, current biological treatments do not adequately address the problem and have difficulty controlling chronic disorders. Major depressive disorder (MDD) is a medical condition that extends beyond low mood or sadness, and its global burden has risen, moving from 19th position in 2016 to 13th in 2019.^3^ Many research studies confirm that the quality of food in the diet is related to the risk of mental health disorders, highlighting the growing importance of nutritional psychiatry in addressing the global burden of mental illness.^5^ Emerging evidence suggests that pro‐inflammatory cytokines may interfere with 5‐hydroxytryptamine (5‐HT) reuptake, potentially worsening its availability in MDD; however, further research is needed to clarify its clinical significance.^6^

The gut, once long‐neglected organ of the human body, is now recognized as a vital regulator of mental and neurological health. There is mounting evidence that the gut microbiome (GM) and microbial dysbiosis contribute to the common mental health and neurocognitive disorders, including anxiety, bipolar disorder, obsessive‐compulsive disorder, and dementia, via the gut‐brain axis (GBA). The increasing prevalence and disability associated with mental health disorders emphasize the need for integrated preventive and therapeutic strategies, as this represents a leading global disability burden, accounting for over 14% of health loss.^7^ GM shapes the central nervous system (CNS) through the GBA, influencing mental health by modulating neurotransmitters, reducing neuroinflammation, and maintaining barrier integrity.^8^ GM dysbiosis also plays a critical role in host neurological disorders through the GBA.^9^ The human gut is a complex ecosystem in which microbial diversity, the immune system, nutrition, and host cells interact closely.^10^ Some strains and their metabolites target the brain via stimulation of the vagus nerve or through the neuroendocrine mechanism. Stress, even at a low level, induces excessive corticosterone and adrenocorticotropic release and deregulates the adrenal axis functions. In addition, pro‐inflammatory cytokines decrease the serotonin level, which leads to psychological disorders, such as anxiety and depression, and a consequent decrease in the host immunity.^11^ Change in GM as a result of consumption of psychobiotics causes a variety of changes, including nervous, endocrine, and immune responses by affecting a person's cognitive, mood, and behavioral reactivity.^12^

Serotonin (5‐HT) is one of the most potent signaling molecules in the gastrointestinal, peripheral, and neuronal systems, playing a key role in regulating energy metabolism. It acts as a hormone influencing glucose homeostasis, lipid metabolism, and thermogenesis, and acts as a trophic factor that helps in the development and maintenance of the enteric nervous system (ENS).^13^ Recent studies have demonstrated a complex interplay between GM and 5‐HT metabolism, with enteric bacteria playing a significant role in producing 5‐HT.^14^ In an age of rising health disorders, mental health‐related disorders have emerged as a major burden around the globe. 5‐HT has the true power to regulate one's mental and gut health, as it plays a crucial role in numerous physiological processes, including mood, cognition, appetite, reward, memory, and the immune system.^15^ This monoamine neurotransmitter exists in plants, animals, and microorganisms. In recent years, microbes have emerged as a promising factor for the synthesis of neurotransmitters.^16^, ^17^, ^18^ In light of growing interest in supporting mental health, the effect of psychobiotics via ingestion could become a valuable treatment for neural dysfunction. This review examines the current understanding of the psychobiotics mechanism in regulating tryptophan (Trp) metabolism and 5‐HT synthesis, as well as the GBA, including vagus nerve signaling, immune modulation, and neuroactive metabolite production, exploring their potential applications in anxiety, depression, cognition, and other neurological disorders.

## 2 GBA: MICROBIOME INFLUENCE ON NEUROCHEMISTRY

The GBA refers to the complex, two‐way communication network that connects the digestive system with the brain, influencing both gut and mental and neurological health. The GM plays a crucial role in shaping neurochemistry by influencing the production and regulation of key neurotransmitters.^19^ Metabolism‐epigenome‐immunity network and GBA modulation by diet impact brain health.^20^ GM produce metabolic products and precursors such as short‐chain fatty acids (SCFA), gamma‐aminobutyric acid (GABA), histamine, Trp, and tyrosine, that are used to produce neurotransmitters through interaction.^21^ GM metabolism provide precursors to Trp metabolism that serves as a central element in the GBA, representing a primary pathway through which GMs contribute to the development of MDD.^22^ Neurotransmitters, including 5‐HT, dopamine, norepinephrine, glutamate, acetylcholine, histamine, and GABA, play a crucial role in both the peripheral nervous system and the CNS. They are divided into excitatory neurotransmitters, inhibitory neurotransmitters, neuromodulators, and neurohormones.^23^ GM has an important role in modulating neurotransmitter production; for instance, *Bifidobacterium dentium* and *Clostridium sporogenes* can contribute to phenylalanine and tyrosine metabolism in the gut. Tyrosine decarboxylase activity in GM, which converts L‐DOPA to dopamine, can reduce the levodopa level in patients with Parkinson's disease.^24^ The diet rich in fermented foods has therapeutic potential for improving intestinal and brain health due to the availability of probiotic microbes. The ENS or intrinsic nervous system controls gastrointestinal function from the esophagus to the anus within its lining, connected to the CNS via autonomic nerves, which operate via reflex, forming the GBA. Studies have reported the influence of the gastrointestinal tract on brain function and its digestive role.^25^ The bidirectional link is mediated by immunological, neuronal, and neuroendocrine pathways, which impact multiple organs and the synthesis and functioning of neurotransmitters. Therefore, disruption in microbiota composition causes dysbiosis that contributes to mental health disorders like depression, anxiety, and other psychiatric conditions.^26^

The GM and its connection with the CNS became a highly interesting subject of study, with the GBA working as a communication network between the gastrointestinal tract and the CNS.^27^ Critically, the GM, comprised of trillions of microorganisms, has the potential to influence host physiology and plays a central role in the regulation of gut health, neurochemistry, and immunomodulatory functions.^28^ GM influences the brain through the neurological system via neural, immune, metabolic, and endocrine pathways, with the influence of neurotransmitters such as 5‐HT.^29^ The GM, which contains approximately 10^15^ microbial cells and more than 22 million microbial genes, produces a variety of metabolites, including SCFAs, neuroactive metabolites, amino acids, gases, bile acid metabolites, and polyamines.^30^ The bidirectional communication initiated by the CNS can modulate gut motility, digestive juice secretion, blood flow, and composition of GM via the gut immune system.^31^ The evidence of this bidirectional communication was further confirmed by using germ‐free animals, which lacked microbiome, and displayed altered stress, anxiety, and depression, highlighting the GM influence on brain health and impact on neurological and psychiatric disorders such as Parkinson′s, Alzheimer's, and autism.^32^, ^33^ Microbial‐produced metabolites shape neurochemical pathways and appear to play a role in regulating behavioral, cognitive, and mood functions. SCFAs, such as butyrate, acetate, and propionate, are produced through the fermentation of fiber, significantly affecting the blood‐brain barrier (BBB), immune cell signaling, and neurotransmitter synthesis.^34^ BBB maintains the homeostasis by isolating brain from bloodstream, functioning as a bidirectional exchange mechanism between brain and microvasculature.^35^ The increase in pro‐inflammatory cytokines increases the permeability of the BBB, leading to psychiatric disorders due to decreased levels of serotonin.^36^ Serotonin is synthesized in the gut by species such as *Clostridium* to affect enterochromaffin (EC) cells but does not freely cross the intact BBB, although neurotransmitter precursors of and 5‐hydroxytryptophan (5‐HT), such as Trp, 5‐HTP, can cross the BBB and subsequently modulate CNS neurotransmitter balance.^37^ 5‐HT is a pivotal neurotransmitter for mood, cognition, and gastrointestinal function; approximately 90–95% 5‐HT is synthesized in the gastrointestinal tract mainly by EC cells in the gut lining, and the remaining 5–10% is produced by serotonergic neurons.^38^ Recent studies suggest that the microbiome can influence host 5‐HT levels by modulating the expression of genes involved in 5‐HT synthesis. GM can regulate microbiome related to virulence, biofilm formation, and motility, within the intestinal ecosystem, and also modulate host 5‐HT synthesis by upregulating expression of tryptophan hydroxylase 1 (*TPH1*) enzyme. Trp is hydroxylated by *TPH1*/*TPH2* to 5‐HTP, decarboxylated by aromatic l‐amino acid decarboxylase (AADC/AAAD) to 5‐HT, then deaminated by monoamine oxidase (MAO) to 5‐hydroxyindole acetaldehyde and oxidized by aldehyde dehydrogenase to 5‐hydroxyindoleacetic acid (5‐HIAA). This host‐microbe interaction can alter 5‐HT levels in the brain by the secretion of microbial secondary metabolites or through the modulation of host gene tryptophan hydroxylase 2 (*TPH*2) and MAO, which regulate metabolism and signaling, ultimately altering brain 5‐HT without crossing the BBB.^39^, ^40^ In a decade‐old study, *Lactobacillus plantarum* PS128 modulated 5‐HT levels in naïve adult mice, which shows an ameliorating effect for anxiety and depression like behavior, as the serotonergic system has been linked to anxiety disorders due to insufficient 5‐HT levels.^41^ Depletion of GM due to antibiotics in adult rats led to significant depressive‐like behaviors and impaired learning, which is associated with dysregulation of 5‐HT in the hippocampus. A placebo‐controlled trial also showed that depressive symptoms significantly improved in the *Lactobacillus plantarum* PS128‐treated patients, with the symbiosis of specific microbial taxa.^42^ These studies have shown that the GM plays a key role in regulating brain activity and cognition. These microbes facilitate communication between the metabolic, peripheral immune, and CNS through the GBA.

## 3 PSYCHOBIOTICS: MICROBIAL MIND‐SHAPERS

In modern mental health prevention, the focus has shifted towards non‐pharmacological strategies to enhance well‐being before symptoms of a disorder. Population‐wide approaches are valuable, targeting at‐risk individuals. For better mood, cognitive and mental health, it is important to balance neurotransmitters as the dysregulation may cause disorders like depression, anxiety, and neuro psychiatric disorders.^43^ Psychobiotics are a specialised group of probiotics—live microorganisms that, when taken in adequate amounts, confer health benefits on the host, particularly by improving aspects of mental health. Psychobiotics can synthesise or modulate neuroactive substances, such as γ‐aminobutyric acid (GABA) and serotonin, and influence host neurological, endocrine, and immune functions through the gut–brain axis.^44^ Psychobiotics influence and release neurotransmitters either through the fermentation of prebiotics or direct production of neuroactive compounds, which interact with the host′s nervous, endocrine, and immune systems, either directly or by altering the GM.^45^ The neurological disorders like Alzheimer′s, Parkinson′s disease, epilepsy, autism spectrum disorder (ASD), depression, and many more, which are associated with an imbalance of neurotransmitters; thus, the supplementation of psychobiotics in an adequate amount is a way to treat neurological disorders.^46^ A detailed summary of psychobiotic interventions and their effects on neurochemical pathways is shown in Table 1. Specific strains of psychobiotics can synthesize or modulate a variety of neuroactive compounds. These include neurotransmitter precursors such as DOPA and 5‐HTP, as well as neurotransmitter‐like molecules, including GABA, 5‐HT, norepinephrine, and acetylcholine. In addition, they produce biogenic amines (e.g., histamine and tryptamine), amino acids (e.g., glutamate, glycine, and tryptophan), and SCFAs including acetate, propionate, and butyrate. Furthermore, microbial metabolism generates gaseous signaling molecules such as nitric oxide (NO) and hydrogen sulfide (H_2_S).^58^ Modulation of host neurotransmitter synthesis and release is done in EC cells. These cells get stimulated by acetate and produce approximately 90% of the body's 5‐HT.^59^ Psychobiotics also release extracellular vesicles (EVs), which are absorbed in the gastrointestinal tract and cross the BBB to transport content to the brain for increasing expression of neurotrophic molecules, improving serotonergic neurotransmission, and supplying astrocytes with glycolytic enzymes to promote neuroprotective mechanisms by regulating epigenetic factors.

**TABLE 1:** Psychobiotic strain targeting mental health disorders by modulating neurochemicals in preclinical and clinical models.

Bacteria strain	Targeted disease	Study design	Study model	Mechanism	Evidence of regulation	Limitations	Reference
*Lactobacillus plantarum*	Depression, Anxiety	Random	Wistar Diabetic rats (*N* = 48)	Modulation of the HPA axis through reduction in oxidative stress; increased antioxidant markers	5‐HT increases; SCFA is produced	The effect of the supplements on the gut microbial composition at weekly intervals over a period of 8 weeks is not evaluated	[47]
*Bifidobacterium breve* CCFM1025	Depression, Anxiety	Random	C57BL/6J Mice (*N* = 40)	Regulation of HPA axis; increased BDNF expression; reduced depression and anxiety‐like behaviors	5‐HT; GABA increase; SCFA is produced	The generated results come from male mice only, unclear metabolite production, lack of knowledge of pathway, and effect duration.	[48]
*Bifidobacterium longum* NCC3001	Anxiety and depression with irritable bowel syndrome (IBS)	double‐blind, placebo‐controlled study	IBS patients (*N* = 44)	Reduced depression; HPA‐axis hyperactivity modulated	5‐HT increases; SCFA is produced	A larger, powered IBS patient with depression trial is needed to confirm results.	[49]
*Bifidobacterium longum* NCC3001	Perinatal mood disorders, Depression and anxiety	double‐blind, placebo‐controlled, randomized, 3‐parallel‐arm study	Pregnant and lactating women (*N* = 184)	Anxiety and depression reduced; Improved sleep quality; no difference in mood outcomes	Trp availability increased, and 5‐HT increased; SCFA is produced	Excluded severe depression/anxiety, limiting observable improvements, and No power calculation; sample size borrowed from vitamin D trial (may underestimate probiotic effects).	[50]
*Bifidobacterium longum* 1714	Stress‐related disorders, anxiety, and cognitive impairment	Placebo‐controlled design	Human (*N* = 22)	Decreased stress and improved memory; the cortisol level is low	Increase in 5‐HT production; increased GABA and SCFA production	Selected healthy population instead of stress‐related population, and small sample size	[51]
*Lactobacillus casei* HY2782, *Bifidobacterium lactis* HY8002	Depression and anxiety	N/A	C57BL/6 mice	Alleviates depression and inflammation	5‐HT production is modulated; SCFA is produced, and reduced inflammatory cytokines	Which probiotic metabolites drive BDNF/serotonin effects unclear	[52]
*Bifidobacterium bifidum* W23, *Bifidobacterium lactis* W52, *Lactobacillus acidophilus* W37, *Lactobacillus brevis* W63, *Lactobacillus casei* W56, *Lactobacillus salivarius* W24, and *Lactococcus lactis* (W19 and W58)	Depression, Rumination, Aggressive cognition	Triple‐blind, placebo‐controlled, randomized, pre‐ and post‐intervention assessment design	Human (*N* = 20)	Normalized HPA‐axis function; decreased rumination and aggressive	5‐HT increased; GABA modulated and reduced cytokines	No inclusion of dietary measures, tested a predominantly female sample, and generalizability to males is uncertain and results are not verified by tests.	[53]
*Lactobacillus pentosus* LPQ, *Lactobacillus. helveticus* Q13, *Lactobacillus. plantarum* M45, *Lactobacillus. casei* 96, *Lactobacillus. plantarum* 05015, *Lactobacillus. plantarum* Q11, *Lactobacillus. plantarum* Q9, *Lactobacillus. rhamnosus* T53, *Lactobacillus. helveticus* Y4, *Lactobacillus. plantarum* 05007	Depression	N/A	RIN‐14B cell culture	Alters TNF and oxidative phosphorylation signaling pathways; secretion of active compounds	Increased secretion of 5‐HTP; modulated Trp	In vitro only	[54]
*Lactobacillus paracasei* CCFM1229, *Lactobacillus rhamnosus* CCFM1228	Depression and anxiety‐related symptoms	Random	C57BL/6J Mice (*N* = 24)	Decreased stress‐like and anxiety‐like behavior; decreased xanthine oxidase activity directly associated with reduced depression and anxiety	5‐HT is modulated via stress pathway normalization; in the brain, xanthine oxidase decreased; increased adenosine deaminase activity	Mode of action and mechanism is not discussed	[55]
*Lactobacillus rhamnosus* GG (ATCC53103)	Depression, anxiety, and stress	Random	Wistar Albino male rat (*N* = 56)	Reduced anxiety‐like behavior; alterations in the expression of GABA in brain regions	Enhanced 5‐HT production; modulated GABA; increased neurotrophic factors	Clinical trials are needed to see the antidepressant effects	[56]
*Bifidobacterium bifidum* BIA‐6, *B. longum* BIA‐8, *B. lactis* BIA‐7, *Lactobacillus acidophilus* T16 *(Mixture with FOS, GOS, inulin)*	Depression	Random	Human (*N* = 75)	Reduced depression; enhanced microbial metabolism and gut–brain signaling	Increase 5‐HT; SCFA is produced; BDNF is increased, and modulation in GABA	self‐report questionnaire, and conventional culturing instead of molecular analysis	[57]

Abbreviations: ATCC, American Type Culture Collection; BDNF, brain‐derived neurotrophic factor; FOS, fructo‐oligosaccharides; GABA, γ‐aminobutyric acid; GOS, galacto‐oligosaccharides; HPA axis, hypothalamic–pituitary–adrenal axis; IBS, irritable bowel syndrome; N/A, not available; SCFA, short‐chain fatty acids; TNF, tumor necrosis factor; Trp, tryptophan; 5‐HT, serotonin (5‐hydroxytryptamine); 5‐HTP, 5‐hydroxytryptophan.

Daily consumption of probiotics modulates the host's immune balance against pathogenic microbiota. The food consumed has prebiotics, bioactive, and other nutritional contents that stimulate the growth of beneficial microbes and are fermented by GM, converted to neuro‐modulatory metabolites, and affect the host immune system.^60^ The concept of the GM is emerging as a metabolic interaction influenced by diet, xenobiotics, genetics, and other environmental factors that affect the host′s absorption of nutrients, metabolism, and immune system. Beyond nutrient digestion and production, the GM also functions as personalized polypharmacy, where bioactive metabolites that our microbes excrete or conjugate may reach systemic circulation and impact all organs, including the brain. Appreciable evidence shows that GM produce diverse neuroactive metabolites, particularly neurotransmitters (and their precursors), which stimulate the local nervous system (i.e., enteric and vagus nerves) and affect brain function and cognition.^61^ Microbial cells possess microbial‐associated molecular patterns (MAMPs) that enable them to communicate with the ENS and the innate immune system. The MAMPs of beneficial gut microbes by triggering pattern‐recognition receptors, stimulate production of cytokines, such as interleukin (IL)‐10 produced by immune cells, suppress pro‐inflammatory cytokines (IL‐1β, tumour necrosis factor alpha [TNF‐α], IL‐6), control excessive immune responses, reduce inflammation, and maintain tissue homeostasis.^18^ SCFA plays a crucial role in immune modulation by activating G protein‐coupled receptors (GPCRs) and inhibiting histone deacetylase (HDAC) activity, which leads to a reduction in inflammation.^62^ The GM metabolizes Trp into various indole derivatives (e.g., indole‐3‐propionic acid [IPA], indole‐3‐acetaldehyde [IAld]) and kynurenine (Kyn) metabolites, many of which have neuroactive and immunomodulatory properties.

Various animal studies have shown that microbial metabolites, including indole, alpha‐tocopherol, tyramine, SCFA, and secondary bile acids, also contribute to 5‐HT production.^63^ Studies have reported that *E. coli* can synthesize Trp due to expression of Trp synthase, which acts as the sole substrate for 5‐HT synthesis. 5‐HT production in the gut acts as a mutualism, as this modulates and enriches the abundance of spore‐forming bacteria in the host gut.^64^ For dopamine synthesis, the involvement of GM involves a preliminary direct enzymatic conversion of precursors. Species like *Enterococcus faecalis* and *Enterococcus faecium* demonstrate decarboxylase activity of the precursor tyrosine through de novo synthesis. AADC activity enables the microbiota to directly convert tyrosine or l‐DOPA into dopamine. Whereas species like *Lactobacillus* and *Bifidobacterium* modulate the dopamine level.^65^

## 4 UNDERSTANDING SEROTONIN AND TRP: KEY FUNCTIONS IN THE BRAIN AND BODY

5‐HT is a metabolite of Trp and a crucial signaling molecule in the intestine, where it regulates gastrointestinal functions, such as peristalsis, vasodilation, and visceral sensitivity, and has been shown to influence gut motility, secretion, inflammation, sensation, and neurogenesis of the ENS.^13^ The 5‐HT in the body comes from the gut, where EC cells synthesize, store, and release it. In two steps, the aromatic amino acid Trp is converted into 5‐HT by mammalian EC cells. Firstly, the dietary l‐Trp is used by EC cells from the bloodstream and converted to 5‐HTP with the help of the enzyme TPH1. In the second step, the 5‐HTP is converted to 5‐HT by aromatic AADC in the cytosol.^66^, ^67^ 5‐HT, synthesized in both the gut and brain, is partially influenced by gut dysbiosis. For instance, the bacterial lipopolysaccharide (LPS) induces the release of inflammatory mediators, triggering systemic inflammation in the gut, which leads to aseptic neuroinflammation.^68^ Serotonin derived from the gut facilitates the development and maintenance of the ENS and resists the inflammation, which is responsible for regulating epithelial secretion and intestinal motility.^69^

5‐HT production is regulated by Trp availability throughout the brain–gut axis, and variations in these levels cause coordination changes in metabolite profiles and gene expression in both systems.^70^ The decrease in Trp and 5‐HT is accompanied by a reduction in the brain′s expression of TPH2, the key enzyme responsible for the production of 5‐HT.^40^, ^71^ Although there is an increase in the expression of SERT (*SLC6A4*) to recapture limited extracellular 5‐HT. Several 5‐HT receptors change, with the 5‐HT1A receptor being more highly expressed, whereas the 5‐HT2A/2 C may decrease or remain stable.^72^ Metabolically, low 5‐HT is accompanied by lower 5‐HIAA and a diversion of Trp into the Kyn pathway, which elevates Kyn and neuroactive metabolites, such as quinolinic acid. In the gut, low Trp and 5‐HT lead to decreased TPH1 expression in EC cells, reduced 5‐HT release, and poorer signaling to motility‐related receptors.^73^ Indoles and SCFA are examples of microbial metabolites that frequently decrease, which lowers TPH1 activity and occasionally affects the expression of barrier‐related genes, including claudin‐1 (*CLD1*) and occludin (*OCLN*).^74^, ^75^

An experimental trial conducted by Liang et al.^76^ on adult male specific‐pathogen‐free Sprague‐Dawley rats to study the administration of *Lactobacillus helveticus* NS8 can improve chronic stress. Experimental group received a strain daily in the drinking water, and a positive control group received the serotonin reuptake inhibitor (SSRI) citalopram. NS8 administration resulted in lower plasma corticosterone and adrenocorticotropic hormone levels and increased levels of IL‐10. Moreover, 5‐HT and norepinephrine levels in the hippocampus are restored. The recovery of 5‐HT may be due to increased Trp availability from the regulation of immune response and hypothalamic–pituitary–adrenal (HPA) axis. Whereas Huang et al.^77^ found that the supplementation of *Lactobacillus paracasei* PS23 could delay age‐related cognitive decline, as the selected model senescence accelerated mouse prone 8 (SAMP8) mice given live bacteria for 16 weeks at a 1 × 10^9^ CFU/mL concentration increased 5‐HT and dopamine. Using a maternal separation (MS) mouse model of early‐life stress, Liao et al. evaluated the effects of live and heat‐killed *Lactobacillus paracasei* PS23 on anxiety‐ and depression‐like behaviors, stress responses, and neuroimmune regulation. Both live and heat‐killed PS23 significantly alleviated anxiety‐ and depression‐like behaviors, reduced serum corticosterone, increased anti‐inflammatory IL‐10 levels, and modulated hippocampal dopaminergic metabolism.^78^ In studies conducted by Tian et al.^48^, ^79^ administration of *B. breve* CCFM1025 in C57Bl/6J mice and MDD humans (*N* = 45) for a clinical study, the strain significantly reduced depression. In animal models, 5‐HT and 5‐HTP levels are increased, whereas in humans, a significant reduction in 5‐HT turnover and upregulation of Trp and 5‐HTP are observed. Overall, the evidence suggests a significant reduction in depressive disorders and an impact on 5‐HT and Trp production. A few clinical trials with lactic acid bacteria (LAB), *Bifidobacterium* strains, or multi‐strain have shown a reduction in depression and anxiety‐like behavior. Fermented milk beverage with *L. casei Shirota* YIT 9029 in a 100 mL bottle with 1 × 10^11^ CFU was given to 49 students (27 males and 22 females) for 8 weeks daily, and was reported to reduce cortisol and stress levels, which are associated with Trp metabolism.^80^ A double‐blind, placebo‐controlled trial on 60 patients,^81^ with *L. plantarum* 299v and SSRI as control for 8 weeks. 30 participants with *L. plantarum* 299v and 30 with placebo underwent assessment by Hamilton Depression Rating Scale, Attention and Perceptivity Test, and biochemical parameters such as Trp, Kyn, TNF‐α, IL‐6, and 3‐hydroxykynurenine were measured. The observed results showed a significant decrease in Kyn concentration in the *L. plantarum* 299v group. The decrease in Kyn concentration can contribute to improved cognitive health in MDD patients.^81^ A multi‐strain study conducted by Steenbergen et al.^53^ on 20 human participants for 28 days proposed an impact on Trp and 5‐HT. A triple‐blind, placebo‐controlled, randomized trial with *B. bifidum W23*, *B. lactis W52*, *L. acidophilus W37*, *L. brevis W63*, *L. casei W56*, *L. salivarius W24*, and *L. lactis (W19 and W58)* was given as a food supplement, 2 grams once a day. In comparison to placebo participants, participants with the probiotic supplement showed reduced sad mood. With reference to clinical studies, depression and anxiety alter microbiota diversity in the gut, which can also be altered by some drugs due to their antibiotic potential. In summary, the preclinical and clinical studies give evidence that probiotic strains like LAB or *Bifidobacterium*, single or multi‐strain, can reduce depression, anxiety, and influence neurotransmitter production such as serotonin, when consumed in food or supplement matrix.^82^

An increase in the levels of Trp and 5‐HT triggers the system to rebalance. Increased 5‐HT in the brain reduces *TPH*2 through feedback inhibition, while downregulating SERT and lowering reuptake.^83^ Receptor dynamics vary correspondingly, with 5‐HT1A receptors tending to downregulate due to desensitization and 5‐HT2A/2C shifting higher in particular neuronal circuits.^84^ Metabolite profiles reveal a decrease in Kyn pathway activity and an increase in 5‐HT and its breakdown product, 5‐HIAA. *TPH1* expression and 5‐HT release are increased in the gut by increased Trp and microbial metabolites, such as butyrate, indole, and tryptamine. While 5‐HT‐responsive receptors, including 5‐HT3 and 5‐HT4, become more active to assist motility, enterocytes may upregulate SERT to preserve luminal balance.^66^, ^85^

## 5 SEROTONIN AND TRP METABOLISM PATHWAYS IN THE GUT

**FIGURE 1:**
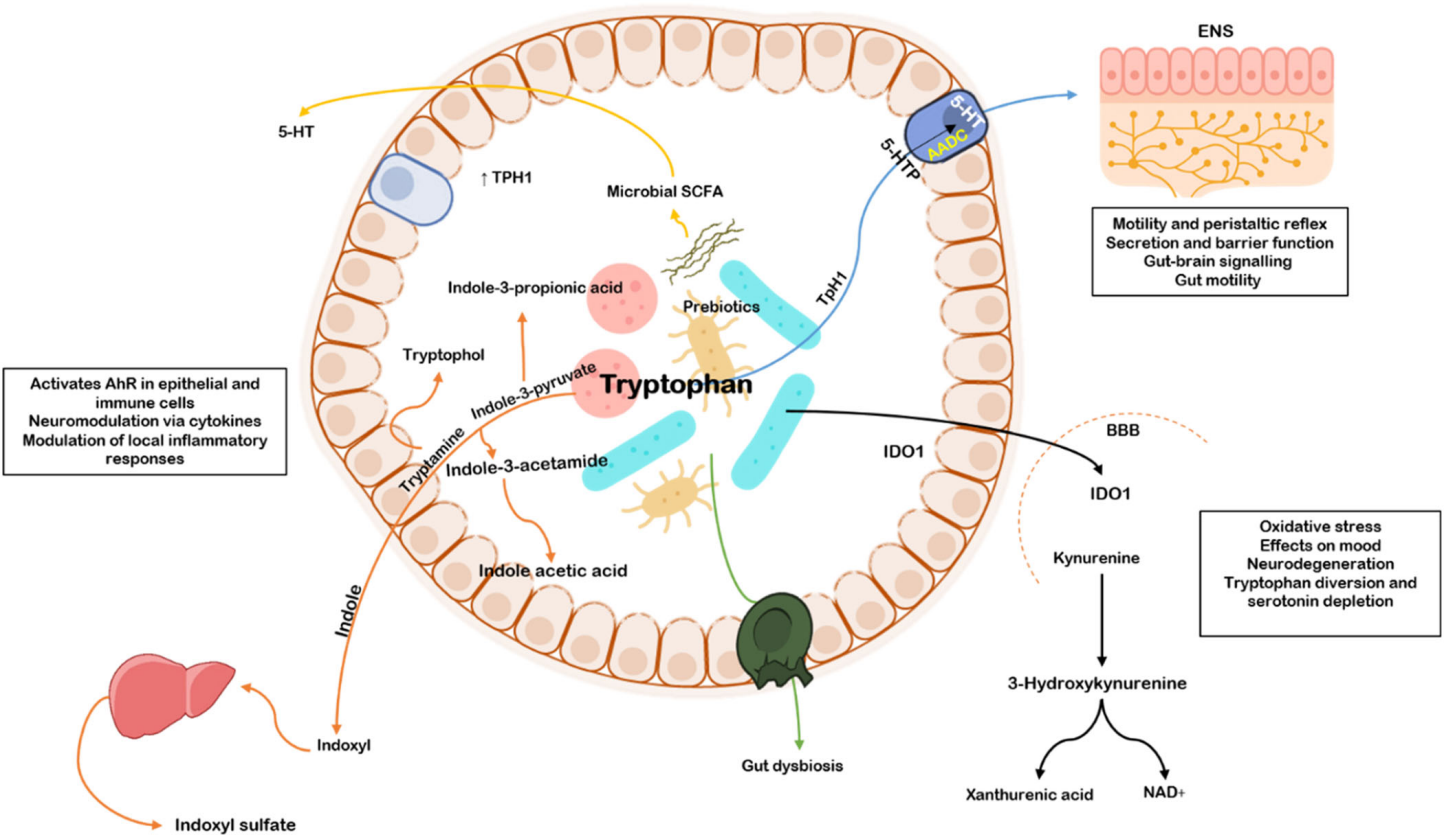
Overview of tryptophan metabolism in the gut lumen. AADC, Aromatic l‐amino acid decarboxylase; AhR, Aryl hydrocarbon receptor; BBB, Blood‐brain barrier; ENS, Enteric nervous system; IDO1, Indoleamine 2,3‐dioxygenase; NAD+, Nicotinamide adenine dinucleotide; TPH1, Tryptophan hydroxylase 1; 5‐HTP, 5‐hydroxy‐tryptophan; 5‐HT, 5‐hydroxy‐tryptamine.

On the cellular level, serotonin is synthesized from Trp, an essential amino acid, via oxidation to 5‐HTP, which requires the rate‐limiting enzyme tryptophan hydroxylase, and decarboxylation of Aromatic‐l‐amino acid to 5‐HT.^86^ Trp metabolism is not limited to the cellular level; the gut microbiota also metabolizes Trp, as it can influence host 5‐HT synthesis and serotonergic signaling. Specific gut microbiota possess an enzyme that synthesizes Trp, which increases 5‐HT in EC cells. An overview of gut microbial tryptophan metabolism and its downstream neurochemical effects is illustrated in Figure 1. The gut microbiota, which confer mental health benefits to the host by producing or modulating neuroactive compounds such as neurotransmitters (GABA, 5‐HT, dopamine, etc.) and precursors, are well known as psychobiotics.^18^, ^87^ Microbial contribution in the production of 5‐HT offers notable advantages, such as continuous production and a short production cycle, because in microbial cells, 5‐HT is synthesized by utilizing the precursor essential amino acid l‐Trp, which is hydroxylated to form 5‐HTP, which is decarboxylated to produce 5‐HT.^15^ Trp is absorbed in the small intestine via the Kyn pathway, the indole pathway, and the 5‐HT pathway. The psychobiotic influence in modulation was associated with alterations in key Trp‐dependent pathways, specifically 5‐HT biosynthesis, the Kyn pathway, and microbial indole metabolism.

### 5.1 Kyn pathway

Trp metabolism occurs along two axes: the 5‐HT axis and the Kyn axis. Where this pathway plays a dual role in the CNS, it can be both harmful and beneficial depending on its concentration.^88^ The Kyn pathway was first discovered in 1853 through the feces of animals fed Trp. The Kyn pathway catabolizes ~99% of Trp, which is not utilized for protein production.^89^ Previously, the Kyn pathway was recognized for its role in producing nicotinamide adenine dinucleotide (NAD+), but current research has shown its important link to brain diseases, depression, cancer, and inflammation. The Kyn pathway contains neuroactive metabolites, including Kyn, kynurenic acid, anthranilic acid, and quinolinic acid, which are implicated in neurological disorders.^90^ The pathway has two branches, either the neurotoxic arm or the neuroprotective arm, which are regulated by the kynurenine 3‐monooxygenase and kynurenine aminotransferase enzymes.^91^ This pathway can be induced by gut microbiota, which indirectly affects serotonergic and glutamatergic pathways.^81^ A reported study^81^ has demonstrated that *Lactobacillus plantarum* 299v treatment improved cognitive performance and decreased Kyn concentration in plasma. Besides the neurotoxic character of Kyn pathway, it shows host–microbe homeostasis by aryl hydrocarbon receptor (AHR) activation, where metabolites of GM and Kyn pathway act as ligands for AHR, influencing immune responses and host‐microbe homeostasis.^90^, ^92^

### 5.2 Indole pathway

Approximately 5% of dietary Trp is metabolized through the indole pathway, which integrates host enzymes and gut microbiota. Tryptophanases, an enzyme of microbiota in the large intestine, convert Trp into indole and its by‐products. The primary metabolites of the pathway include indole‐3‐acetic acid (IAA), indole‐3‐aldehyde (IAID), indole‐3‐acetaldehyde (IAAId), indole‐3‐lactic acid (ILA), indole‐3‐propionic acid (IPA), indole acrylic acid (IA), tryptamine, and skatole, which are produced via protein fermentation due to a lack of carbohydrate.^93^ Indoles are also produced by enzymes in the liver via l‐amino acid oxidase, IL‐4‐induced‐1, and xenobiotic enzymes.^94^ Indole and its derivatives regulate inflammation, CNS, and autoimmune response through various cellular activities mediated by AHR, which can regulate neurological disorders.^95^ Indoles and their derivatives are produced by both Gram‐positive bacteria, such as *Lactobacillus* and *Bifidobacterium*, and Gram‐negative bacteria, including *Escherichia coli, Bacteroides*, and *Clostridium*. The recent study done by Qian et al.^96^ has shown that psychobiotic *Bifidobacterium breve* increased the level of ILA in blood, a derivative of indole in humans and mice models, by identifying aromatic lactate dehydrogenase (*Aldh*) gene expression responsible for making ILA, resulting in an antidepressant effect.

### 5.3 Serotonin biosynthesis pathway in EC cells

EC cells produce a major portion of the 5‐HT concentration, and are the most populated enteroendocrine cells in the intestine.^97^ EC cells produce 95% of the total body 5‐HT through stimulus‐dependent mechanisms and coordinate with two main forms of communication in the gastrointestinal tract lining, namely luminal and basolateral signaling.^98^ GM maintains a symbiotic relationship with the host gut for various biological functions by influencing 5‐HT synthesis. Bacterial species like *Streptococcus*, *E. coli*, *B. subtilis* R0179,^99^ and*Corynebacterium* are capable of synthesizing Trp via Trp synthase, which provides additional support to EC cells for the synthesis of 5‐HT.^100^ 5‐HT is primarily synthesized from the amino acid Trp through the rate‐limiting enzyme *TPH1* present in EC cells for regulating signaling via the GBA and *TPH2* in serotonergic neurons.^101^ *TPH1* and *TPH2* convert Trp to L‐5‐HTP, which is converted into 5‐HT by aromatic AADC. After decarboxylating 5‐HT in EC cells, it is transported into vesicles via vesicular monoamine transporter 1 (VMAT1) located at the basolateral membrane of EC cells. Serotonin is stored in EC cells until it gets stimulated, and is released by exocytosis into the surrounding.^102^ The regulation of 5‐HT by host and microbiota via GBA bidirectional communication depends on the availability of Trp, gut bacteria like *Lactobacillus*, *Enterococcus*, *Clostridium*, *Staphylococcus*, and *Pseudomonas* that modulate host 5‐HT level directly and indirectly.^94^

### 5.4 SCFA signaling pathway

GM plays a crucial role in metabolizing various nutrient contents and by‐products, such as SCFA, derived from complex, non‐digestible polysaccharides through fermentation.^103^ Acetate(C2): propionate(C3): butyrate(C4) are SCFAs found in a molar ratio of 60:25:15 in the colon. A study conducted by Reigstad et al.^104^ has shown that SCFAs produced by GM promote serotonin production in EC cells. SCFAs produced by GM get absorbed into colonocytes via H+‐dependent monocarboxylate transporter (MCTs) or sodium‐dependent MCTs and bind to GPCRs. Microbiota‐derived SCFAs also modulate the serotonergic system by regulating the function and expression of SERT and various other 5‐HT receptor‐like 5‐HT1A, 5‐HT2B, and 5‐HT7, which control extracellular 5‐HT availability.^103^

### 5.5 Immunomodulatory pathways

5‐HT functions as a neurotransmitter and potent immunomodulatory molecule, operating at the intersection of the neural and immune signaling systems. An immunomodulatory pathway governs 5‐HT production, modulating mental health through the GBA.^105^ Pro‐inflammatory cytokines, such as IFN‐γ, TNF‐α, IL‐1β, and IL‐6, redirect Trp metabolism from 5‐HT synthesis to the Kyn pathway and activate the HPA axis, increasing cortisol synthesis.^106^ 5‐HT can enter the Kyn pathway via conversion into 5‐hydroxykynuramine by indoleamine 2,3‐dioxygenase (IDO1).^107^ IDO activation depletes local Trp while simultaneously generating kyn metabolites that regulate immunoregulatory properties. Psychobiotics restore balance between 5‐HT and Kyn metabolism, reduce neuroinflammation, and support mental health outcomes.

Psychobiotics exert their beneficial effects on mental health by inducing potent anti‐inflammatory responses, which reduce pro‐inflammatory cytokine production by inhibiting IDO activation, thereby preserving Trp availability for 5‐HT synthesis.^108^ In a study conducted by Li et al.,^109^ multi‐strain treatment containing *L. helveticus* R0052, *L. plantarum* R1012, and *B. longum* R0175 in a chronic mild stress (CMC) mouse model of stress and depression related disorders, the bacteria have reversed CMC and reduced IFN‐γ, TNF‐α, and IDO‐1 levels. Psychobiotics modulate the differentiation and function of various immune cells, thereby shifting the balance from pro‐inflammatory to anti‐inflammatory, restoring intestinal integrity, producing beneficial metabolites such as SCFAs, indole derivatives, and directly upregulating 5‐HT by synthesizing the *TPH1* enzyme and modulating vagal nerve and HPA axis signaling.^110^ Strain‐specific differences in serotonin production, metabolism, and signaling across in vitro, animal, and clinical models are detailed in Table 2.

**TABLE 2:** Psychobiotics modulating serotonin pathways: Actions, mediators, and indications.

Strain	Origin of strain	Model/detection technique	Action on serotonin/metabolites	Other potentials	References
*Lactobacillus pentosus* S4	Stingless bee honey	Millet fermentation, then detection by HPLC (Zorbax Eclipse‐AAA columns)	High Trp accumulation/enhanced Trp availability, which is a precursor of 5‐HT	High probiotic potential, strong acid and salt tolerance	[111]
*Lactobacillus pentosus* LPQ1	Fermented food	RIN‐14B cell culture	Promote secretion of 5‐HTP	Alters the TNF and oxidative phosphorylation signaling pathways	[54]
*Lactobacillus amylovorus, Limosilactobacillus reuteri*, and *Streptococcus alactolyticus*	Piglet gut and feces	Cell line, ELISA, and HPLC	Increase 5‐HT concentration in the colon, upregulation of *TPH1* expression, and promotes 5‐HT production in IPEC‐J2 cells	Downregulation of IDO1, upregulation of acetate, lactate, and free fatty acid receptor 3(FFAR3)	[112]
*Escherichia coli Nissle* 1917	—	C57BL/6 mice	Increased extracellular 5‐HT level in mouse ileal tissue; Modulation of *TPH1*	**‐**	[113]
*Lactobacillus paracasei* CCFM1229 & *Lactobacillus rhamnosus* CCFM1228	human feces or fermented food	C57BL/6J mice	Increased brain 5‐HT concentrations; Regulates purine metabolism; decreases xanthine oxidase activity; increases liver adenosine deaminase activity	Alleviation of depression‐ and anxiety‐related symptoms in mice	[55]
*Lactiplantibacillus plantarum* LZU‐J‐TSL6	Jiangshui (fermented food)	C57BL/6J mice	Restores 5‐HT level in the mouse hippocampus	Increase GABA content; increased abundance of *Bacteroides* and *Muribaculum*	[114]
*Lactiplantibacillus plantarum* BXM2	***—***	UPLC‐MS/MS	Increased content of indole‐3‐lactic acid and di‐/tri‐peptides.	Enhanced flavor and nutritional quality of mulberry juice; confers additional health potential.	[115]

Abbreviations: ELISA, enzyme‐linked immunosorbent assay; FFAR3, free fatty acid receptor 3; GABA, γ‐aminobutyric acid; HPLC, high‐performance liquid chromatography; IDO1, indoleamine 2,3‐dioxygenase‐1; TNF, tumor necrosis factor; TPH1, tryptophan hydroxylase‐1; Trp, tryptophan; UPLC‐MS/MS, ultra‐performance liquid chromatography‐tandem mass spectrometry; 5‐HT, serotonin (5‐hydroxytryptamine); 5‐HTP, 5‐hydroxytryptophan.

## 6 MICROBIOTA‐DERIVED SEROTONIN FROM FERMENTED FOOD AND IMPACT ON MENTAL HEALTH

Psychobiotics represent an emerging therapeutic frontier for mental health disorders. Recent research by Moretti et al.^13^ demonstrated that GM *Limosilactobacillus mucosae* and *Ligilactobacillus ruminis* can synthesize 5‐HT in vitro by decarboxylation of 5‐HTP and raise the level of fecal 5‐HT, colonic neuronal density, and 5‐HT‐immunoreactive neurons in serotonin‐deficient germ‐free mice, and maintain ENS, which further controls bowel motility, and can control irritable bowel syndrome (IBS). The finding also suggests GM can synthesize serotonin. Research was first conducted in zebrafish to analyze the microbiota‐GBA, demonstrating that dietary administration of *Lactobacillus rhamnosus* IMC 501 increased the expression of 5‐HT signaling genes.^116^ 5‐HT production is significantly affected by the production of SCFAs by GM; thus, microbiota‐induced 5‐HT production in the colon depends on the concentration of SCFAs and *TPH1* expression. The same study has demonstrated a metabolic relationship between SCFAs and 5‐HT, where strain *L. reuteri* amended depressive‐like behaviuor in three treatments by ameliorating the 5‐HT level in the colon. As a promising solution for mental health disorders psychobiotics are emerging as promising solution *Bifidobacterium breve* CCFM1025 has the ability to metabolize Trp to give 5‐HT that regulates mood disorders, with the decreased hippocampal ILA level in the depressed mouse model is restored.^96^

The non‐treatable neurodegenerative Parkinson's and Alzheimer's disease develop the α‐synucleinopathy condition of nerve cell death, and a decline in dopamine production.^117^ For managing symptoms of Parkinson's, levodopa is used, which shows several side effects in patients, such as serotonergic dysfunction and alteration of the GABAergic system. Regarding concerns about GABA and 5‐HT dysfunction due to levodopa,^118^ demonstrated the application of psychobiotics bacteria and screened them for tyrosine decarboxylase, GABA, SCFAs, and 5‐HT production using thin‐layer chromatography, Fourier transform infrared spectroscopy, spectrophotometric methods, and 16S rRNA sequencing. These analyses were performed to evaluate GABA and 5‐HT production by the isolated bacteria and identify bacterial strains capable of producing GABA, 5‐HT, and SCFAs, thereby allowing targeted control of Parkinson's and engaging the GBA. A symbiotic intervention using *Limoscilactobacillus fermentum* K73, high‐oleic palm oil, and whey in Colombian children with and without ASD led to increased synthesis of butyric acid and microbial serotonin synthesis with association of increased abundance of probiotic bacteria, including *Lactobacillus, Streptococcus*, and *Anaerostipes*, while reducing Bacteroides.^119^

The application of bioactive compounds to improve mental health disorders has garnered attention due to their potential to modulate and regulate physiological and neurological processes.^120^ Fermented food offers an affordable dietary intervention that comprises beneficial microbes and bioactive molecules with neuroactive potential.^121^ As a large amount of 5‐HT is produced by the EC cell of the gut, host diet, GM, and microbial metabolites shape the 5‐HT concentration in the gut.^122^ There is growing research focusing on serotonin secretion in response to the influence of fermented food, as 5‐HT biosynthesis leads to the production of neuroactive metabolites, such as indole and Kyn. Against this backdrop, fermented kefirs are administered to mice, resulting in increased serotonin turnover in the colon compared to unfermented milk controls.^123^ A double‐blind, randomized, placebo‐controlled clinical trial involving 110 patients with MDD received either placebo, probiotic (*L. helveticus* and *B. longum*), or prebiotics (*Galactooligosaccharide*). The probiotic group reduced Kyn pathway activity, lowered the Beck Depression Inventory level, and normalized 5‐HT synthesis by modulating Trp metabolism, thereby improving symptoms of MDD. In contrast, the prebiotic showed no major effects.^124^

## 7 FUTURE PROSPECTS, CHALLENGES, AND LIMITATIONS OF THE STUDY

The imbalance of neurotransmitters has been the main cause of mental health disorders. Anxiety and depression are both related to dysregulation of 5‐HT in the synaptic cleft.^125^ The response to stress hormones can be reversed by the intervention of psychobiotics, which can be taken as dietary supplements or added to food products to improve gut and brain health.^126^ Neurodevelopmental disorder is associated with the dysregulation of 5‐HT and GABA, which affects 1 in 100 children.^127^ Research has suggested that ingestion of *Lactobacillus, Bifidobacterium*, and *Streptococcus* rebalances the GM composition and firmicutes in children.

However, psychobiotics with promising preclinical evidence, some of the significant gaps still persist, particularly regarding 5‐HT production and gut and mental health support. The first and most important challenge is how closely related probiotics work differently in vivo. Moving forward to human application, it is mandatory to carefully interpret animal behavioral study data.^128^ While the unclear molecular basis, inconsistency in bioactive potential, and cross‐talk with the host cell, psychobiotics derived from humans (16%) and food (16%) mainly belong to the genera *Lactobacilli* (45.5%) and *Bifidobacterium* (29%), which are responsible for producing neuromodulatory metabolites.^129^ The critical gap is how peripherally produced 5‐HT and Trp cross the BBB to influence the CNS,^51^ as 95% of systemic Trp is metabolised via the Kyn pathway, which is initiated by the enzymes tryptophan 2,3‐dioxygenase (TDO) and indoleamine 2,3‐dioxygenase (IDO).^130^

Although psychobiotics may influence peripheral serotonin biosynthesis and tryptophan metabolism, the exact pathways by which they impact CNS function remain unclear, as serotonin itself does not cross the BBB. The lack of standardized dosing, duration, and potential dependency for better support of mental health has insufficient research for the long‐term effect.^131^ The microbiota–gut–brain axis involves complex bidirectional communication that is directly or indirectly influenced by various factors, including diet, timing of intervention, and stress state.

It has been reported by many studies that intestinal tract bacteria have the potential to regulate anxiety and depression disorders. Current studies demonstrate that bacteria isolated from food sources have the capability to produce neurotransmitters and their related metabolites. Notable examples include *Lactiplantibacillus plantarum* LZU‐J‐TSL6, isolated from Chinese fermented food Jiangshui. The strain has the capability to produce 3.838 g/L of GABA and restore the level of 5‐HT in anxiety‐disordered mice, as well as restore the levels of brain‐derived neurotrophic factor, glial fibrillary acidic protein, and 5‐HT markers.^114^ Studies have found that LAB promotes 5‐HTP secretion and has a good ability to relieve depressive symptoms. *Lactobacillus* (L.) *pentosus* LPQ1 isolated from fermented food has the strongest 5‐HTP secretion‐promoting effects.^62^ Despite the identification of the gene encoding aromatic amino acid hydroxylase and decarboxylase in bacteria, direct 5‐HT production has not yet been detected from the majority of food bacteria.^13^, ^132^, ^133^

Psychobiotics are viewed as uniquely suited to treat the multi‐modal syndromes and can be the gastric distress‐free alternative but future research should investigate if psychobiotics increase available Trp by inhibiting its breakdown along Kyn pathway. The primary challenge in current psychobiotics remains unclear if microbial shifts drive better mental health or improved mental health serves as a stabilizer of gut homeostasis. Psychobiotics represent as favorable alternative to Trp supplementation in depressive disorders but lack deep mechanistic. This evidence demonstrates that metabolites from psychobiotic strains support mental health; however, the factors driving these metabolites are poorly understood. Future research should prioritize randomized controlled trials with standardized protocols, mechanistic studies combined with metabolomics analyses, and investigations of strain‐specific effects in diverse populations. Despite promising findings linking psychobiotics and 5‐HT regulation, current clinical evidence remains limited. Human studies suggest that psychobiotics can improve mood, sleep, and behavioral styles, and influence markers associated with 5‐HT metabolism, but the specific mechanisms remain unclear. Due to strain‐specific variation in neuroactive metabolites production, future studies should also investigate the potential of multispecies psychobiotic formulation. Many studies discussed different psychological variables and outcomes, leading to a lack of uniformity of effect on serotonin‐mediated mental health outcomes. These limitations restrict the application in medicine, as clinical outcomes regarding 5‐HT modulation for mental health support are inconsistent and insufficient. Therefore, a large‐scale, standardized, long‐term clinical trial is required for therapeutic approaches and to establish the precise role of psychobiotics in 5‐HT modulation and mental health support.

## 8 CONCLUSION

5‐HT influences various physiological processes, like gut motility, inflammation, sleep, appetite, and even neurogenesis within the ENS. An imbalance in 5‐HT levels develops neurological and neuropsychiatric disorders, like depression, anxiety, autism, IBS, and many more. The current global scenario reveals the burden of mental health disorders and creates a need for therapeutic alternative than pharmacology and traditional treatment because of limitations and side effects due to their side. Psychobiotics are an emerging and promising way to enhance mental and overall health by modulating the balance of neurotransmitters in the gut and modulating the GBA. The key bidirectional highway in this action is the vagus nerve, which helps to transmit signals between the gut and the brain, causing modulation of the CNS. This communication involves a complex set of molecules that influence memory, mood, the balance between inhibitory and excitatory processes, and synaptic plasticity. The primary mechanism by which psychobiotics support the 5‐HT pathway is by modulating its precursor, that is, Trp metabolism. 5‐HT, largely produced by EC cells in the gut, is derived from Trp.

The application of psychobiotics in functional food holds potential for safe alternative to enhance 5‐HT production by modulating GM. Food enriched with psychobiotics can offer a natural and accessible way to improve overall human health. Fermented food is a rich source of probiotic microbiome, and can be an ideal candidate for delivering psychobiotics that modulate neurotransmitter balance. By incorporating a specific strain known for its ability to influence 5‐HT or other neurotransmitter pathways, one can improve experience by reducing anxiety and depression and supporting overall mental and cognitive health. However, the field is still evolving, and several gaps still need to be addressed. While preclinical studies show promising results, more rigorous clinical trials are needed to confirm the efficacy, optimal dosages, and long‐term benefits of specific psychobiotic strains in humans. A large‐scale, well‐designed randomized clinical trial integrating microbiome, metabolomics, immunological, and mechanistic studies is required. Nonetheless, psychobiotics represent an encouraging avenue for integrated mental health treatment, offering a novel, diet‐based strategy to support neurological and immunological well‐being through the power of gut microbiota.

## AUTHOR CONTRIBUTIONS

Nidhi Mandloi, Aarzoo, and Bhim Pratap Singh contributed to conceptualization, methodology, investigation, writing‐original draft preparation, and visualization. Chakkaravarthi Saravanan and Heera Ram are responsible for writing, reviewing, editing, and supervision. All authors have read and approved the final manuscript.

## CONFLICT OF INTEREST STATEMENT

The authors declare no conflicts of interest.

## ETHICS STATEMENT

Not applicable.

## Data Availability

The sources from which data and content were taken for this review are already mentioned in the **reference section**.
